# Spatial-temporal characteristics and prediction of ischemic heart disease burden in the working population aged 15 to 59 years in China

**DOI:** 10.3389/fcvm.2025.1643717

**Published:** 2025-10-21

**Authors:** Yuanhang Zhang, Ruyi Du, Xiaoshuai Zhang, Tiankai Li, Lei Gao

**Affiliations:** ^1^ School of Health Management, Harbin Medical University, Harbin, China; ^2^ The First Affiliated Hospital of Harbin Medical University, Harbin, China

**Keywords:** ischemic heart disease, burden of disease, trend of change, age-period-cohort model, forecasting

## Abstract

**Objective:**

Based on GBD 2021 data, to analyze the disease burden of ischemic heart disease (IHD) in the Chinese population.

**Methods:**

The mortality rate, incidence rate, prevalence rate and Disability-Adjusted Life Years (DALYs) data of IHD in the Chinese population aged 15–59 from 1990–2021 were extracted and age-standardized. Joinpoint regression was used to analyze the trend. The Age-Period-Cohort model (APC) decomposed age, period and cohort effects, and the Arima model predicted the disease burden.

**Results:**

From 1990–2021, the age-standardized mortality rate (ASMR) and the DALYs rate (ASDR) decreased by an average annual percentage change (AAPC) of −0.76 (95% CI: −0.94 to −0.57) and −0.71 (95% CI: −0.87 to −0.55), respectively. The age-standardized prevalence (ASPR) and incidence (ASIR) increased by 0.53 (95% CI: 0.49–0.57) and 0.78 (95% CI: 0.64–0.91), respectively. The decline in ASMR and ASDR in women was greater than that in men, while the increase in ASPR and ASIR was more significant. The risks of death, DALYs and disease onset increase with age. The risk of onset first increased and then decreased. The risks of death and DALYs fluctuated and decreased over time. The risks of death and DALYs peaked from 2002–2006, and incidence risk peaked from 2007–2011. The risk of disease onset increased with the progress of the birth cohort, while the risks of death and DALYs decreased. In the next 15 years, ASIR, ASMR and ASDR of IHD in China will generally show a downward trend. The decline in ASMR and ASDR in women will be greater than that in men, but ASIR in women will increase and ASIR in men will decrease.

**Conclusion:**

There are gender differences in the IHD burden among the working population in China. Health education and early screening for middle-aged and elderly men should be strengthened, and lifestyle intervention should be carried out for middle-aged and young women.

## 1 Introduction

Ischemic Heart Disease (IHD), also known as Coronary Heart Disease (CHD) (1), is a series of clinical syndromes caused by coronary atherosclerosis leading to vascular stenosis or blockage, and subsequently resulting in insufficient blood supply to the myocardium. Its common clinical manifestations include angina pectoris, myocardial infarction, breathing difficulties, etc. In severe cases, it can lead to heart failure, cardiogenic shock and even sudden death (2). IHD is one of the leading causes of death worldwide and has long ranked among the top causes of death and disability globally, posing a significant threat to public health. According to the World Health Organization (WHO), in 2021, the number of deaths caused by IHD worldwide was approximately 8.99 million, resulting in a loss of 180 million disability-adjusted life years (DALYs), accounting for a considerable proportion of the global total burden of disease (3, 4). In China, IHD has become the second largest public health challenge after lung cancer. Its main risk factors include hypertension, high LDL cholesterol, excessive sodium intake, smoking, drinking, lack of exercise, obesity, etc. (5). With the rapid development of the social economy and the transformation of lifestyle, the disease burden of IHD is still constantly rising, and the age of onset shows an obvious trend of getting younger. This phenomenon is particularly significant in developing countries (6, 7). For instance, in recent years, the urbanization process in China has accelerated, and people's lifestyles have gradually become Westernized. The widespread existence of risk factors such as high-fat and high-sugar diets and lack of exercise has further increased the risk of developing IHD. In addition, environmental factors such as air pollution are also closely related to the onset of IHD, further increasing the complexity of the disease burden (8, 9). However, at present, most of the research on IHD focuses on the elderly population, and there are relatively few specialized studies on the working-age population. People in this age group are the main force in social and economic activities, and their health conditions directly affect social productivity and economic development. Therefore, an in-depth study of the epidemic trend of IHD among the working population aged 15–59 in China is of great significance for formulating public health policies, optimizing the allocation of medical resources, and improving the efficiency of disease prevention and control. Based on this, this study, based on the Global Burden of Disease 2021 (GBD 2021) database, focuses on the epidemic trend of IHD among the population aged 15–59 in China. It conducts in-depth analysis from multiple dimensions such as incidence rate, mortality rate, and DALYs rate, aiming to provide a scientific basis for the formulation of public health policies, assist in optimizing the allocation of medical resources, and improve the efficiency of disease prevention and control.

## 2 Data and methods

### 2.1 Data sources

This study was based on the GBD 2021 database, which covers data on 369 diseases and 88 risk factors in 204 countries and regions worldwide from 1990–2021 (10, 11).When GBD 2021 data is used to generate estimates of the global burden of disease, it faces inherent limitations brought about by inconsistent input data quality and model dependence. The original data shows significant heterogeneity in terms of geographical coverage, diagnostic criteria and reporting completeness, especially in low-income and middle-income regions and in some years, there are problems of data sparsity or low quality. To address these challenges, GBD adopts standardized modeling strategies, including the use of DisMod-MR 2.1 for epidemiological data integration and correction, the utilization of Cause of Death Ensemble Model (CODEm) to enhance the robustness of death estimation, and the interpolation and smoothing of missing data with the aid of spatio-temporal Gaussian regression to generate continuous and comparable estimation results. To quantify the above uncertainties, GBD provides a 95% uncertainty interval (UI) based on posterior sampling for all estimates to comprehensively reflect data sampling errors, model differences, and uncertainties from other sources. Annual data of IHD in China from 1990–2021 were used. In the GBD data classification, IHD was classified as a tertiary cause, which was classified as a non-communicable disease in the first level and a cardiovascular disease in the second level (12). IHD was defined according to I20–I25 codes of the International Classification of Diseases, 10th Revision (ICD-10), which covers angina, myocardial infarction, and chronic ischemic heart disease. Data are available at the Global Health Data Exchange gbd results Tool (https://vizhub.healt hdata.org/gbd-results/).

### 2.2 Research metrics

The data of IHD in China from 1990–2021 were extracted from the GBD 2021 database. “Ischemic heart disease”, “Deaths”, “Prevalence”, “Incidence” and “DALYs” were selected for disease burden index, “China” was selected for region, and “1990–2021” was selected for year. Sex selection “to Both” and “Male” and “Female”, age choose “15–19”, “20–24,”, “25–29”, “30–34”, “35–39”, “40–44”, “45–49”, “50–54”, “55–59”, a total of nine age. These indicators can reflect the disease burden of IHD from different perspectives. Among them, Deaths reflect the level of death caused by IHD and are an important indicator for measuring the severity of the disease. Prevalence reflects the number of IHD patients at a specific time point and can demonstrate the epidemic range of the disease. The Incidence rate reflects the number of new cases and is a key indicator for evaluating the trend of disease occurrence. DALYs take into account both the loss of healthy life years due to prevalence and the loss of life years due to premature death, and can comprehensively measure the overall burden of prevalence. The rough data obtained were directly standardized with reference to the world population in GBD 2021 standard to eliminate the influence of age structure differences among different populations, ensure the comparability of the data, and provide an accurate and reliable data basis for subsequent analysis.

### 2.3 Statistical methods

#### 2.3.1 Joinpoint regression model

The study adopted the Joinpoint 4.9.0.0 software developed by the National Cancer Institute of the United States to analyze the changes in the standardized incidence, prevalence, mortality and DALYs of IHD in China. The Joinpoint regression model is a statistical method based on piecewise linear regression. Its core principle is to identify the inflection points of the model, divide the long-term trend of the disease into multiple segments, and describe the epidemic characteristics of the disease with straight line fitting for each segment. This model can effectively capture the changing trends of disease burden indicators in different time periods, identify the turning points of the trends, and thereby more accurately reflect the changing patterns of disease epidemics (13). In this study, the maximum inflection point number was set to 5, and the optimal model was selected through the automatic optimization function of the software to ensure the fitting effect and explanatory ability of the model. The dynamic changes of the IHD indicator were evaluated by calculating the Annual Percent Change (APC) and the Average Annual Percent Change (AAPC). Among them, a positive AAPC or APC value indicates an increase in the IHD index, while a negative value indicates a decrease.

#### 2.3.2 Age-period-cohort model analysis

The age-period-cohort (APC) model is a statistical method widely used in the analysis of disease epidemic trends and etiology. It can decompose the long-term changes in disease incidence, mortality, and DALYs rates into effects in three dimensions: Age, Period, and Cohort (14). Among them, the age effect reflects the changes in an individual's disease risk as they age, embodying the process of physiological aging and natural development. The period effect refers to external factors that have an impact on all age groups within a certain period of time, such as advancements in medical technology, public health policies, and changes in the social environment. The cohort effect captures the differences in disease risks within the same birth cohort due to shared environmental exposure, social and cultural background, etc. The APC model has an inherent identification problem, that is, due to the linear relationship of “queue = period—age” among age, period and queue variables, the model cannot obtain a unique solution. To address this issue, this study employs the widely recognized Intrinsic Estimator (IE) method for parameter estimation (15). By introducing a set of appropriate constraints, IE can obtain unique and unbiased estimates without relying on external assumptions, thereby ensuring the stability and interpretability of effect estimates. In the modeling process of this study, the age was divided into 9 5-year interval groups. To meet the requirements of the IE method for equidistant period structure, the data from 1990–1991 were excluded in this study, and the research period was divided into six consecutive fifth-grade intervals. On this basis, birth cohorts were generated through “period-age” calculation and 14 birth cohorts were generated accordingly to systematically evaluate the impact of the cohort effect on the changes in the disease burden of IHD among the working population in China. APC data analysis and visualization of the model by analyzing the national cancer institute online platform (https://analysistools.cancer.gov/apc) and the Origin of 2024 software implementation.

#### 2.3.3 Autoregressive integrated moving average (ARIMA) model

The ARIMA model is a classic time series analysis method, widely applied in fields such as economics, finance, and meteorology. This model transforms non-stationary sequences into stationary processes through three parameters: autoregressive order (*p*), difference order (*d*), and moving average order (*q*), and captures their autocorrelation features by using autoregressive and moving average structures (16). It can effectively describe long-term trends, seasonal variations, and random fluctuations in time series. This study first preliminarily identifies the values of *p* and *q* based on the truncation and tailing characteristics of the autocorrelation function (ACF) and the partial autocorrelation function (PACF), and determines the d value that makes the sequence stable through differential processing. Further, the Akaike information Criterion (AIC) and the Bayesian information Criterion (BIC) are adopted for model selection to balance the goodness of fit and model complexity. To evaluate the robustness and predictive performance of the model, the Ljung-Box test was conducted on the residuals to verify their non-autocorrelation, and the normality of the residuals was tested through the Q-Q plot. Meanwhile, the time series cross-validation method is adopted to divide the training set and the validation set, and the out-of-sample prediction accuracy is evaluated based on the root mean square error (RMSE) and the mean absolute percentage error (MAPE). The final model was selected to predict the changing trends of relevant indicators of ischemic heart disease among the working population aged 15–59 in China from 2022–2036. All analyses and visualizations are implemented on the R4.2.2 platform.

## 3 Results

### 3.1 The burden of ischemic heart disease

In 2021, the number of deaths from ischemic heart disease among people aged 15–59 in China was 214.05 thousand, and the standardized mortality rate was 17.31 per 100,000. Compared with the number of deaths in 1990, it increased by 58.71%, and the mortality rate decreased by 20.41%. From the perspective of gender differences, the number of deaths and standardized mortality rates of men in each year were higher than those of women. In terms of DALYs, the number of disabled adjusted cases in 2021 was 8,838.70 thousand, and the standardized DALYs rate was 748.86 per 100,000, an increase of 50.31% compared with the number of disabled adjusted cases in 1990, while the standardized DALYs rate decreased by 18.97%. Among them, the number of disabled people and the standardized DALYs rate of males in each year were both higher than those of females. In terms of the patient population, the number of patients in 2021 was 15,491.67 thousand, and the standardized prevalence rate was 1,199.51 per 100,000, increasing by 151.48% and 18.10% respectively compared with 1990. Among them, the number of patients and the standardized prevalence rate of men in each year were both higher than those of women. In terms of the affected population, the number of cases in 2021 was 1,859.46 thousand, and the standardized incidence rate was 149.42 per 100,000, increasing by 152.17% and 26.36% respectively compared with 1990. Among them, the number of cases and standardized incidence rates of men in each year were both higher than those of women see Table 1.

**Table 1:** The burden of ischemic heart disease among the 15–59 age group in China from 1990–2021.

Indicator	Year	Prevalence		Incidence		Deaths		DALYs	
		Quantity (1,000, 95% CI)	Standardized rate (/ 100,000, 95% CI)	Quantity (1,000, 95% CI)	Standardized rate (/ 100,000, 95% CI)	Quantity (1,000, 95% CI)	Standardized rate (/ 100,000, 95% CI)	Quantity (1,000, 95% CI)	Standardized rate (/ 100,000, 95% CI)
Male	1990	3,734.40 (2,931.10–4,678.16)	1,176.58 (925.42–1,470.80)	463.45 (277.02–707.64)	142.31 (85.77–216.86)	84.13 (67.89–102.28)	25.81 (20.78–31.46)	3,689.62 (3,002.06–4,456.10)	1,104.09 (896.38–1,337.52)
	2021	9,017.49 (6,893.20–11,644.99)	1,377.08 (1,049.58–1,784.85)	1,122.41 (697.00–1,679.31)	178.18 (109.72–267.20)	158.20 (118.26–206.31)	25.48 (19.23–33.17）	6,559.41 (4,983.02–8,487.41）	1,103.55 (844.06–1,420.83)
	Rate of change	141.47	17.04	142.19	25.21	88.04	−1.28	77.78	−0.05
Female	1990	2,425.93 (1,895.88–3,050.83)	837.23 (656.35–1,049.71)	273.92 (156.60–426.13)	91.64 (52.93–142.01)	50.74 (40.35–63.36)	17.28 (13.76–21.57)	2,190.67 (1,745.20–2,726.21)	726.52 (579.44–903.22)
	2021	6,474.18 (4,952.48–8,420.86)	1,018.59 (775.08–1,331.10)	737.05 (430.56–1,135.55)	119.86 (69.60–184.67)	55.85 (41.80–72.71)	8.88 (6.66–11.62)	2,279.29 (1,729.11–2,964.36)	380.16 (288.01–495.27)
	Rate of change	166.87	21.66	169.07	30.79	10.07	−48.61	4.05	−47.67
Both	1990	6,160.32 (4,830.63–7,737.73)	1,015.67 (798.48–1,272.32)	737.37 (434.60–1,127.44)	118.25 (70.32–180.28)	134.87 (115.22–155.80)	21.75 (18.57–25.15)	5,880.29 (5,034.71–6,775.54)	924.22 (790.42–1,065.67)
	2021	15,491.67 (11,854.34–20,099.50)	1,199.51 (914.52–1,562.40)	1,859.46 (1,133.63–2,806.02)	149.42 (90.41–225.93)	214.05 (170.23–265.20)	17.31 (13.81–21.40)	8,838.70 (7,119.18–10,837.93)	748.86 (604.71–916.03)
	Rate of change	151.48	18.10	152.17	26.36	58.71	−20.41	50.31	−18.97

### 3.2 Age distribution characteristics

**Figure 1:**
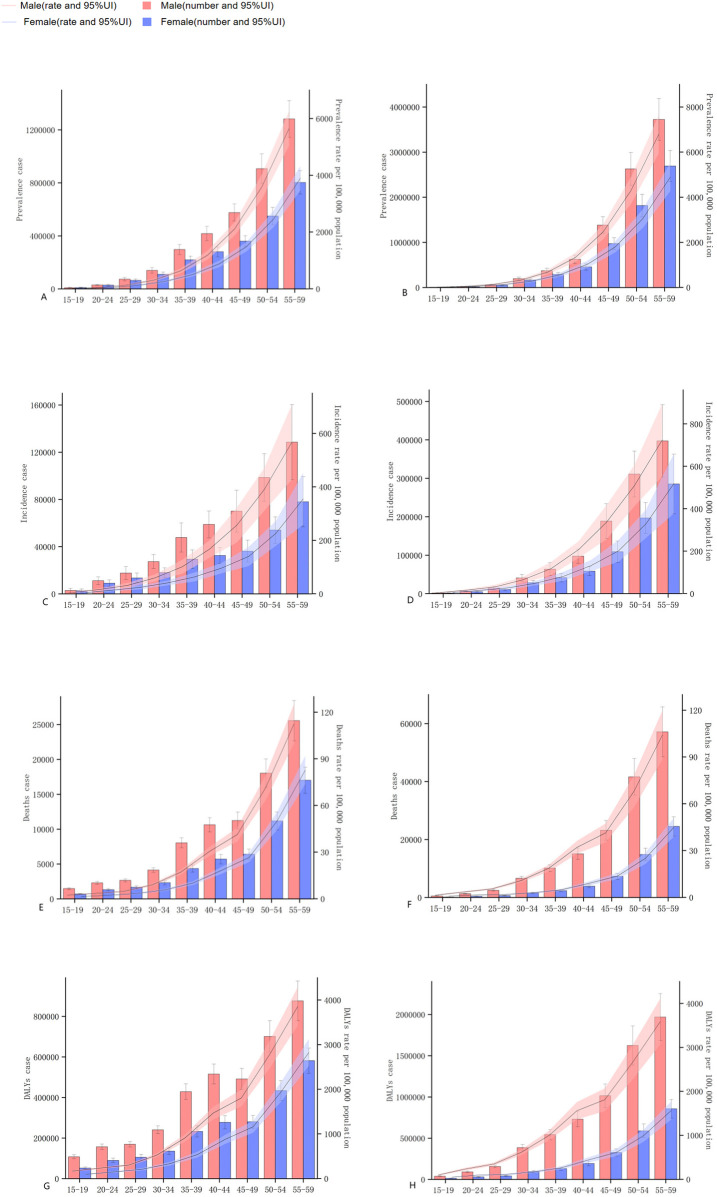
The changes of the prevalence, incidence, mortality, and the number and rate of DALYs of ischemic heart disease in China from 1990–2021. **(A,C,E,G)** represent the quantities and rates of prevalence, incidence, mortality, and DALYs in 1990. **(B,D,F,H)** represent the quantities and rates of prevalence, incidence, mortality, and DALYs in 2021.

Overall, among the population aged 15–59 in China, the prevalence, incidence, death and DALYs of ischemic heart disease increase with age. In 2021, the number of prevalence, incidence, death and DALYs were all higher than those in 1990, the rates of prevalence and incidence increased, but the rates of death and DALYs decreased. Men were higher than women in terms of the number and rate of prevalence, onset, death and DALYs in each age group. Compared with 1990, the differences in DALYs and the number of deaths among men and women of different age groups further widened in 2021. The prevalence and incidence of ischemic heart disease among people aged 15–59 in China increased in 2021 compared with 1990, while the mortality rate and DALYs rate decreased. Compared with 1990, the differences in DALYs and mortality rates between men and women of different age groups further widened in 2021 see Figure 1.

### 3.3 Joinpoint regression analysis on time trends of ischemic heart disease burden

#### 3.3.1 Time trend of age-standardized prevalence rate (ASPR)

**Figure 2:**
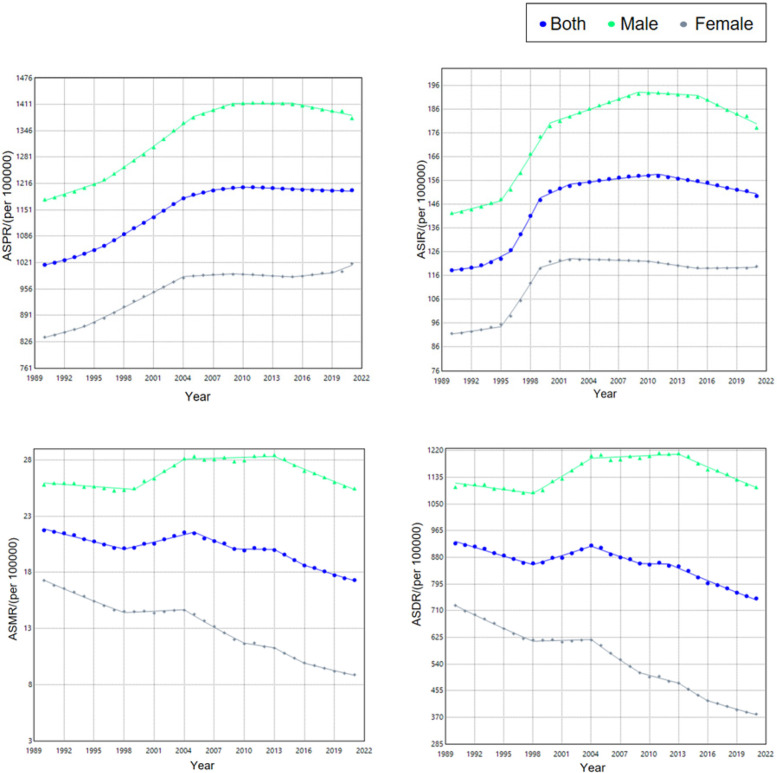
Trend of burden of ischemic heart disease among Chinese population aged 15–59 years, 1990–2021.

From 1990–2021, the ASPR of ischemic heart disease among Chinese adults aged 15–59 rose significantly, with an AAPC of 0.53 (95% CI: 0.49–0.57). Segmented analysis revealed a modest decline from 2010–2021 (APC = −0.07, 95% CI: −0.09 to −0.05), whereas all earlier intervals displayed an upward trajectory, most steeply between 1996 and 2004 (APC = 1.32, 95% CI: 1.29–1.35). When stratified by sex, both men and women experienced sustained increases; however, the pace among women exceeded that among men (AAPC 0.62 vs. 0.53) see Tables 2, 3 and Figure 2.

**Table 2:** Annual average trend of ischemic heart disease burden in Chinese population aged 15–59 years from 1990–2021.

Indicators	Both		Male		Female	
	AAPC (95% CI)	*P* value	AAPC (95% CI)	*P* value	AAPC (95% CI)	*P* value
ASPR	0.53 (0.49–0.57)	<0.01	0.53 (0.48–0.58)	<0.01	0.62 (0.57–0.67)	<0.01
ASIR	0.78 (0.64–0.91)	<0.01	0.76 (0.68–0.84)	<0.01	0.86 (0.79–0.93)	<0.01
ASMR	−0.76 (−0.94–−0.57)	<0.01	−0.07 (−0.18–0.04)	0.21	−2.14 (−2.36–−1.92)	<0.01
ASDR	−0.71 (−0.87–- 0.55)	<0.01	−0.04 (−0.14–0.06)	0.42	−2.08 (−2.32–−1.83)	<0.01

AAPC, annual average percent change.

**Table 3:** Trends of ischemic heart disease in Chinese population aged 15–59 years from 1990–2021.

Indicators	Both		Male		Female	
	Year	APC (95% CI)	Year	APC (95% CI)	Year	APC (95% CI)
ASPR	1990–1993	0.60 (0.4–0.74)	1990–1996	0.68 (0.58–0.77)	1990–1994	0.78 (0.64–0.93)
	1993–1996	0.90 (0.65–1.16)	1996–2005	1.35 (1.30–1.41)	1994–2004	1.34 (1.30–1.38)
	1996–2004	1.32 (1.29–1.35)	2005–2009	0.58 (0.32–0.85)	2004–2009	0.13 (−0.01–0.26)
	2004–2007	0.55 (0.30–0.80)	2009–2015	0.01 (−0.11–0.12)	2009–2015	−0.12 (−0.22–−0.02)
	2007–2010	0.20 (−0.04–0.45)	2015–2021	−0.35 (−0.45–−0.25)	2015–2019	0.24 (0.02–0.47)
	2010–2021	−0.07 (−0.09–−0.05)			2019–2021	0.93 (0.42–1.45)
ASIR	1990–1993	0.46 (0.04–0.89)	1990–1995	0.75 (0.53–0.97)	1990–1995	0.66 (0.53–0.79)
	1993–1996	1.71 (0.88–2.55)	1995–2000	4.09 (3.77–4.40)	1995–1999	6.01 (5.71–6.31)
	1996–1999	5.62 (4.75–6.50)	2000–2009	0.77 (0.67–0.87)	1999–2002	1.03 (0.45–1.61)
	1999–2002	1.27 (0.42–2.12)	2009–2015	−0.12 (−0.31–0.07)	2002–2010	−0.11 (−0.18–−0.03)
	2002–2011	0.30 (0.21–0.39)	2015–2021	−1.06 (−1.21–−0.90)	2010–2015	−0.49 (−0.67–−0.32)
	2011–2021	−0.53 (−0.59–−0.47)			2015–2021	0.05 (−0.03–0.15)
ASMR	1990–1998	−1.06 (−1.20–−0.92)	1990–1999	−0.24 (−0.40–−0.08)	1990–1998	−2.25 (−2.42–−2.08)
	1998–2005	1.03 (0.81–1.26)	1999–2004	2.03 (1.48–2.58)	1998–2004	0.29 (−0.06–0.66)
	2005–2009	−1.77 (−2.41–−1.12)	2004–2013	0.09 (−0.08–0.27)	2004–2010	−3.70 (−4.06–−3.34)
	2009–2013	−0.06 (−0.74–0.62)	2013–2021	−1.36 (−1.54–−1.17)	2010–2013	−1.20 (−2.78–0.39)
	2013–2016	−2.38 (−3.75–−0.99)			2013–2016	−4.21 (−5.67–−2.71)
	2016–2021	−1.52 (−1.86–−1.18)			2016–2021	−2.28 (−2.60–−1.96)
ASDR	1990–1998	−1.03 (−1.20–−0.86)	1990–1998	−0.37 (−0.56–−0.18)	1990–1998	−2.11 (−2.32–−1.89)
	1998–2004	1.11 (0.82–1.40)	1998–2004	1.65 (1.34–1.96)	1998–2004	0.14 (−0.21–0.51)
	2004–2009	−1.23 (−1.61–−0.86)	2004–2013	0.11 (−0.03–0.27)	2004–2009	−3.68 (−4.11–−3.24)
	2009–2012	−0.06 (−1.43–−1.32)	2013–2021	−1.14 (−1.38–−0.89)	2009–2013	−1.67 (−2.47–−0.86)
	2012–2021	−1.57 (−1.76–−1.38)			2013–2016	−4.06 (−5.98–−2.10)
					2016–2021	−2.18 (−2.77–−1.59)

APC, annual percentage change.

#### 3.3.2 Temporal trend of age-standardized incidence rate (ASIR)

From 1990–2021, the ASIR of ischemic heart disease among Chinese adults aged 15–59 rose significantly, with an AAPC of 0.78 (95% CI: 0.64–0.91). Segmented analysis revealed a decline from 2011–2021 (APC = −0.53, 95% CI: −0.59 to −0.47), whereas all earlier intervals displayed an upward trajectory, most steeply between 1996 and 1999 (APC = 5.62, 95% CI: 4.75–6.50). When stratified by sex, both men and women experienced sustained increases; however, the pace among women exceeded that among men (AAPC 0.86 vs. 0.76).

#### 3.3.3 Time trend of age-standardized mortality rate (ASMR)

From 1990–2021, the ASMR for ischemic heart disease among Chinese adults aged 15–59 declined significantly, with an AAPC of −0.76 (95% CI: −0.94 to −0.57). Segmented analysis revealed a transient upward shift during 1998–2005 (APC = 1.03, 95% CI: 0.81–1.26), whereas all other intervals displayed a downward trajectory, steepest in 2013–2016 (APC = −2.38, 95% CI: −3.75 to −0.99). Throughout the entire period, the decrease among women outpaced that among men (AAPC −2.14 vs. −0.07).

#### 3.3.4 Time trend of age-standardized DALYs rate (ASDR)

From 1990–2021, the ASDR for ischemic heart disease among Chinese adults aged 15–59 fell significantly, with an AAPC of −0.71 (95% CI: −0.87 to −0.55). Segmented analysis revealed a transient rise during 1998–2004 (APC = 1.11, 95% CI: 0.82–1.40), whereas all other intervals displayed a downward trajectory, steepest in 2012–2021 (APC = −1.57, 95% CI: −1.76 to −1.38). Throughout the entire period, the decrease among women outpaced that among men (AAPC −2.08 vs. −0.04).

### 3.4 Age-period-cohort model

#### 3.4.1 Age-period-cohort analysis of incidence

**Figure 3:**
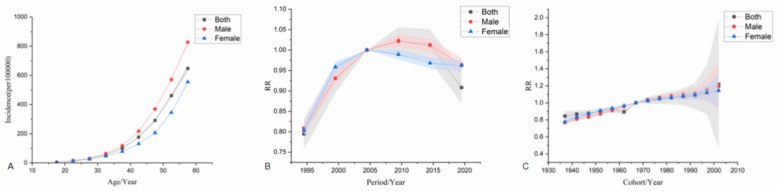
Age-Period-Cohort analysis model of incidence. **(A)** is the age effect, **(B)** is the period effect, **(C)** is the cohort effect.

The age effect shows that the risk of ischemic heart disease increases with age across the country, with a higher risk in men than in women. The period effect showed that, taking 2002–2006 as the reference period (RR = 1.00), the national incidence risk from 1992–2021 presented an increasing trend first and then a decreasing trend, and the period with the highest risk was 2007–2011 (RR = 1.02, 95% CI: 0.95–1.09). Among them, the risk of disease onset in both male and female populations increased first and then decreased. The period with the highest risk for men was from 2007–2011 (RR = 1.02, 95% CI: 0.99–1.05). The period with the highest risk for women was from 2002–2006 (RR = 1.00). The cohort effect showed that, taking the birth cohort from 1967–1971 as a reference (RR = 1.00), the risk of disease onset in the national population showed an upward trend. The birth cohort from 2002–2006 had the highest risk (RR = 1.21, 95% CI: 0.44–3.38), among which the risks in both male and female populations showed an upward trend. The male population born between 2002 and 2006 had the highest risk (RR = 1.19, 95% CI: 0.76–1.87). The female population born between 2002 and 2006 had the highest risk (RR = 1.14, 95% CI: 0.81–1.60) see Figure 3.

#### 3.4.2 Age-period-cohort analysis of death

**Figure 4:**
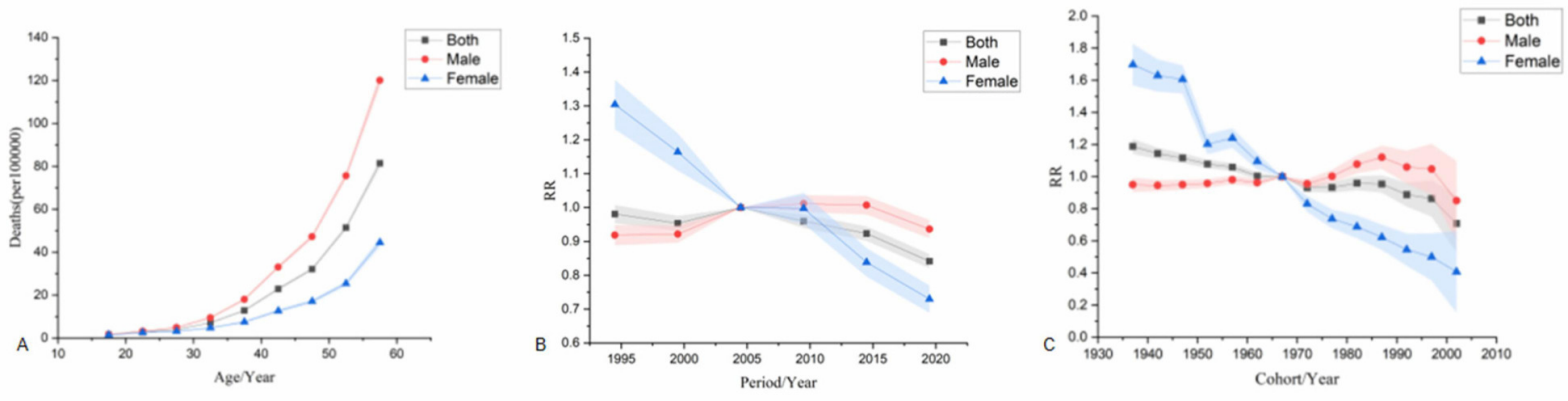
Age-Period-Cohort analysis model for death. **(A)** is the age effect, **(B)** is the period effect, **(C)** is the cohort effect.

The age effect shows that from 1992–2021, the mortality risk of the national population increased with age for both men and women, and the mortality risk for men was significantly higher than that for women. The period effect showed that taking 2002–2006 as the reference period (RR = 1.00), the national mortality risk showed a fluctuating downward trend, and the highest risk period was from 2002–2006 (RR = 1.00). Among males, the mortality risk increased first and then decreased slowly, with the highest risk period from 2007–2011 (RR = 1.01, 95% CI: 0.96–1.06). The mortality risk of females showed a downward trend, and the highest risk period was from 1992–1996 (RR = 1.30, 95% CI: 1.17–1.46). The cohort effect showed that the mortality risk of the national population showed a fluctuating downward trend when taking the 1967–1971 birth cohort as reference (RR = 1.00), and the risk was highest in the 1937–1941 birth cohort (RR = 1.19, 95% CI: 1.10–1.28). In terms of gender, the risk of death in males increased first and then decreased, and the risk of death in males born during 1987–1991 was the highest (RR = 1.12, 95% CI: 0.99–1.27). The mortality risk in females showed a fluctuating downward trend, with females born during 1937–1941 having the highest mortality risk (RR = 1.70, 95% CI: 1.46–1.97) see Figure 4.

#### 3.4.3 Age-period-cohort analysis of DALYs

**Figure 5:**
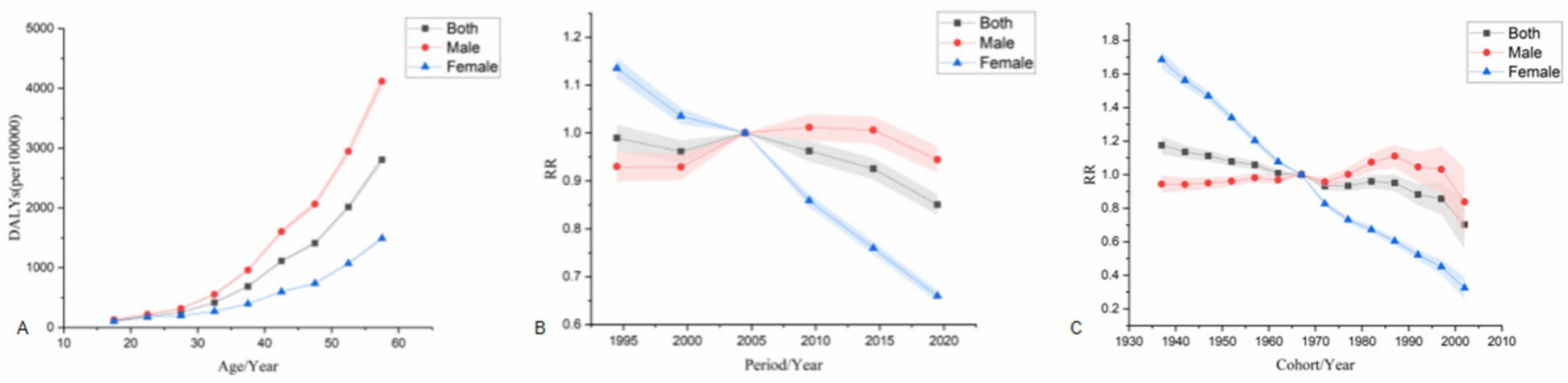
Age-Period-Cohort analysis model for DALYs. **(A)** is the age effect, **(B)** is the period effect, **(C)** is the cohort effect.

The age effect shows that the risk of DALYs in ischemic heart disease increases significantly with age, and the risk of DALYs in men is significantly higher than that in women.The period effect showed that the reference period was 2002–2006 (RR = 1.00). From 1992–2021, DALYs showed a fluctuating downward trend, with the highest risk period from 2002–2006 (RR = 1.00). Among males, the DALYs increased first and then decreased, with the highest risk period from 2007–2011 (RR = 1.01, 95% CI: 0.96–1.07). The highest risk period of DALYs in females was from 1992–1996 (RR = 1.14, 95% CI: 1.09–1.18). The cohort effect showed that the risk of DALYs in the whole country showed a fluctuating downward trend when using the 1967–1971 birth cohort as a reference (RR = 1.00), and the risk of DALYs was highest in the 1937–1941 birth cohort (RR = 1.18, 95% CI: 1.08–1.28). The risk of DALYs in males increased first and then decreased, with the highest risk seen in males born during 1987–1991 (RR = 1.11, 95% CI: 0.99–1.25). The risk of death in females showed a fluctuating downward trend, with the highest risk in females born during 1937–1941 (RR = 1.79, 95% CI: 1.69–1.89) see Figure 5.

### 3.5 Prediction of disease burden in the next 15 years

#### 3.5.1 ASIR prediction

**Figure 6:**
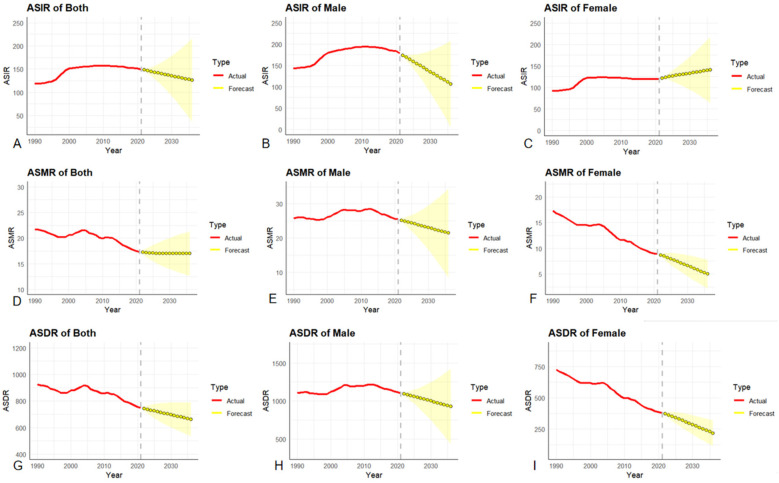
**(A–C)**: ARIMA prediction model for ASIR, **(D–F)**: ARIMA prediction model for ASMR, **(G–I)**: ARIMA prediction model for ASDR.

The ASIR among the Chinese working population is on the decline, decreasing from 149.42/100,000 in 2021–125.72/100,000 in 2036, with an overall decline rate of 15.86%. After conducting a gender stratification analysis of the prediction results, we found that there are fundamental differences in the ASIR change trends between men and women. The male population is on a downward trend, dropping from 178.18/100,000 in 2021–105.88/100,000 in 2036, representing a decline of 40.58%. In sharp contrast, the ASIR of the female population rose from 119.86/100,000 in 2021–140.64/100,000 in 2036, representing a growth of 17.34% see Figure 6.

#### 3.5.2 ASMR prediction

The ASMR of ischemic heart disease among the national working population showed a downward trend, decreasing from 17.31/100,000 in 2021–16.98/100,000 in 2036, with an overall decline rate of 1.91%. Among them, the ASMR rate of ischemic heart disease showed a downward trend in both men and women,but there were gender differences in the extent of the decline. The ASMR of the male population decreased from 25.48/100,000 in 2021–21.44/100,000 in 2036, with a decline rate of 15.86%. The ASMR of the female population decreased from 8.88/100,000 in 2021–4.99/100,000 in 2036, with a decline rate of 43.81%. The decline in ASMR in women is 2.8 times that in men.

#### 3.5.3 ASDR prediction

The ASDR of ischemic heart disease among the working population in China showed a downward trend, from 748.86/100,000 in 2021–661.45/100,000 in 2036, with an overall decline rate of 11.67%. The prevalence of ischemic heart disease showed a downward trend in both males and females. The ASDR of the male population decreased from 1,103.55/100,000 in 2021–927.87/100,000 in 2036, with a decline rate of 15.92%. The ASDR of the female population decreased from 380.16/100,000 in 2021–217.18/100,000 in 2036, with a decline rate of 42.87%. The decline in ASDR in women is 2.7 times that in men.

## 4 Discussion

Over the past three decades or so, the ASMR and ASDR of IHD among the working population aged 15–59 in China have shown a significant downward trend. This reflects that China has achieved positive results in the prevention and treatment of cardiovascular diseases, especially in reducing premature deaths and the loss of healthy life years. This achievement is attributed to the continuous advancement of medical technology (such as more efficient revascularization techniques and drug treatment plans), the wide popularization of health education for all, and the in-depth implementation of the national-level chronic disease prevention and control strategy (17, 18). However, in contrast to the decline in mortality rate, the ASPR and ASIR of IHD have continued to rise, indicating that the prevalence of the disease is still expanding and the number of new cases is constantly increasing. This seemingly contradictory phenomenon is closely related to China's profound social and economic transformation and the rapid changes in people's lifestyles (19, 20). With the rapid development of industrialization, urbanization and globalization, the dietary structure of residents has become increasingly Westernized. Diets high in fat, sugar and salt have become widespread. At the same time, physical activity levels have significantly declined, and bad behavioral habits such as smoking and drinking are widespread. These factors have jointly promoted the prevalence of metabolic risk factors such as obesity, hypertension and diabetes. This thereby increases the risk of developing IHD (21, 22).

It is worth noting that there are significant gender differences in each indicator of the disease burden of IHD. Specifically, although the absolute levels of ASMR and ASDR in the male population are higher than those in women, the extent of their decline is significantly smaller than that in women. Meanwhile, the upward trend of ASPR and ASIR in men is even more significant. This pattern of difference may be driven by multiple factors together. Firstly, there are gender differences in the exposure levels of behavioral risk factors. The latest survey data confirm that the smoking rate among Chinese men is consistently and significantly higher than that among women (23), and smoking is an independent risk factor for IHD, whose harm has been confirmed by a large number of studies (24, 25). Secondly, there are gender differences in the accessibility and utilization behavior of health services. Studies have shown that compared with women, men tend to seek medical services later and have lower compliance with preventive health check-ups. This may lead to delayed diagnosis of IHD, insufficient or non-standard treatment, thereby affecting the final prognosis (26–28). In addition, social and psychological factors, such as men usually facing higher work pressure and social expectations, may also play a certain role (29). In contrast, premenopausal women have a relatively lower risk of IHD due to the protective effect of endogenous estrogen on the cardiovascular system (30, 31). However, it is worth noting that this study observed that the increase rate of ASIR in women has exceeded that in men, suggesting that as women's social roles change and life pressure increases, this traditional protective advantage may be being weakened.

In addition to gender differences, the disease burden of IHD shows significant regional variations. Existing studies have shown that the incidence and mortality risks of IHD gradually increase from east to west. The age-related incidence and mortality rates are the highest in the western region, while they are relatively lower in the central region (32). This is closely related to the imbalance in economic and social development and the uneven distribution of medical resources. Economically developed regions are usually more effective in controlling risk factors, while regions with limited resources face greater challenges (33, 34). At the urban-rural level, although some data in rural areas show a lower incidence rate, due to poor accessibility of medical resources, limited disease management capabilities and weak health awareness among residents, IHD patients are often diagnosed later and have a lower treatment rate, ultimately resulting in significantly higher mortality and disability-adjusted life years than those in urban areas (35, 36). To this end, it is necessary to enhance zero-level prevention (such as the promotion of healthy lifestyles) and secondary prevention (such as the construction of chest pain centers and the management of chronic cardiovascular diseases) among people in the western regions and rural areas in order to alleviate health inequality.

With the help of the APC model, we further analyzed the deep-seated driving forces behind the changes in disease burden. The age effect shows that for both men and women, the risk of death, onset and DALYs from IHD increases sharply with age, and the risk level for men is systematically higher than that for women in all age groups. This is not only related to the natural decline of physiological functions and the reduced reserve capacity of the cardiovascular system brought about by aging (37, 38), but may also be associated with the earlier occurrence of atherosclerosis in men and the gender differences in lipid metabolism and immune inflammatory responses (39, 40). This indicates that public health intervention measures must focus on middle-aged and elderly men, a high-risk group, and enhance their health management and early screening. The period effect reflects the impact of the external environment on all age groups. Data shows that after 2005, the mortality rate and DALYs risk of IHD entered a plateau or even a downward channel, which indicates that the series of chronic disease prevention and control policies, medical technology popularization and public health projects implemented by the country over the past two decades have begun to show effects (41, 42). The cohort effect reveals the influence of the birth generation. The risk of death and DALYs in the later birth cohort shows a downward trend, which reflects that with the development of the social economy, the overall nutritional status, medical accessibility and health awareness of the nation have been fundamentally improved (43).

The prediction based on the ARIMA model provides a forward-looking basis for the prevention and control strategies of IHD in China. In the next 15 years, the ASIR, ASMR and ASDR of IHD in China will show an overall downward trend. Among them, the decline in ASMR and ASDR in women is significantly higher than that in men, suggesting that women may benefit more obviously in health behavior compliance, utilization of primary medical services, and public health interventions such as prenatal and postnatal care and major disease screening (26, 44). However, it should be noted that the incidence of ASIR in women is on the rise, indicating that although China has made substantial progress in the clinical treatment and advanced disease management of IHD, there are still deficiencies in the control of source risks and primary prevention in the population. The female group is particularly confronted with complex challenges such as the superimposition of multiple comorbidity risks related to aging and the increasing social psychological pressure (45, 46). Future prevention and control policies must shift their focus forward, formulate more targeted and gender-sensitive proactive prevention strategies, and through lifestyle intervention and early risk management of the population, curb the growth trend of IHD incidence and achieve a comprehensive reduction in the disease burden.

This study has certain limitations. Firstly, its data is derived from the model estimates of GBD. Although it has been strictly corrected, it still cannot completely avoid the uncertainties brought about by model assumptions and the quality of input data. Secondly, stratified analysis of urban-rural and regional differences has not been conducted, making it difficult to deeply reveal the intrinsic heterogeneity of disease burden. Furthermore, the failure to integrate multiple risk factors for joint analysis has limited the in-depth explanation of the reasons for the changes in disease burden from the perspective of risk exposure.

## 5 Conclusions

Special attention should be paid to the elderly male group. Health education should be strengthened to enhance their awareness of IHD and self-management ability, and targeted work stress relief and mental health support should be provided. At the same time, efforts should be made to promote early screening and intervention for IHD, enhance the accessibility and quality of medical services, and ensure that high-risk groups receive timely and effective diagnosis and treatment. Among the female population, it is necessary to further optimize health management strategies and strengthen lifestyle interventions to reduce exposure to risk factors. In addition, in light of China's actual conditions, efforts should be made to promote multi-departmental collaboration, coordinate multiple factors such as the social environment, behavioral patterns, and medical resources, continuously improve the prevention mechanism for high-risk groups, and focus on the long-term management, rehabilitation, and comprehensive prevention and control of IHD to reduce the disease burden, safeguard the health of the labor force, and promote sustainable social and economic development.

## Data Availability

The original contributions presented in the study are included in the article/Supplementary Material, further inquiries can be directed to the corresponding author.
